# Circulating tumor DNA is readily detectable among Ghanaian breast cancer patients supporting non-invasive cancer genomic studies in Africa

**DOI:** 10.1038/s41698-021-00219-7

**Published:** 2021-09-17

**Authors:** Samuel Terkper Ahuno, Anna-Lisa Doebley, Thomas U. Ahearn, Joel Yarney, Nicholas Titiloye, Nancy Hamel, Ernest Adjei, Joe-Nat Clegg-Lamptey, Lawrence Edusei, Baffour Awuah, Xiaoyu Song, Verna Vanderpuye, Mustapha Abubakar, Maire Duggan, Daniel G. Stover, Kofi Nyarko, John M. S. Bartlett, Francis Aitpillah, Daniel Ansong, Kevin L. Gardner, Felix Andy Boateng, Anne M. Bowcock, Carlos Caldas, William D. Foulkes, Seth Wiafe, Beatrice Wiafe-Addai, Montserrat Garcia-Closas, Alexander Kwarteng, Gavin Ha, Jonine D. Figueroa, Paz Polak, the Ghana Breast Health Study Team, Robertson Adjei, Lucy Afriyie, Anthony Adjei, Florence Dedey, Victoria Okyne, Naomi Ohene Oti, Evelyn Tay, Adu-Aryee, Angela Kenu, Obed Ekpedzor, Marion Alcpaloo, Isaac Boakye, Bernard Arhin, Emmanuel Assimah, Samuel Ka-Chungu, Joseph Oppong, Ernest Osei-Bonsu, Margaret Frempong, Emma Brew Abaidoo, Bridget Nortey Mensah, Samuel Amanama, Prince Agyapong, Debora Boateng, Ansong Thomas Agyei, Richard Opoku, Kofi Owusu Gyimah, Lisa Newman, Louise A. Brinton, Maya Palakal, Jake Thistle, Michelle Brotzman, Shelley Niwa, Usha Singh, Ann Truelove, Richard Biritwum

**Affiliations:** 1 Department of Biochemistry and Biotechnology, Kwame Nkrumah University of Science and Technology, Kumasi, Ghana; 2 Department of Oncological Sciences, Icahn School of Medicine at Mount Sinai, New York City, New York, USA; 3 Molecular and Cellular Biology Program, University of Washington, Seattle, WA USA; 4 Division of Public Health Sciences, Fred Hutchinson Cancer Research Center, Seattle, WA USA; 5 Division of Cancer Epidemiology and Genetics, National Cancer Institute, Bethesda, MD USA; 6 Korle Bu Teaching Hospital, Accra, Ghana; 7 Komfo Anokye Teaching Hospital, Kumasi, Ghana; 8 Research Institute of the McGill University Health Centre, Montréal, QC Canada; 9 University of Ghana, Accra, Ghana; 10 Department of Population Health Science and Policy, Icahn School of Medicine at Mount Sinai, New York City, New York, USA; 11 Tisch Cancer Institute, Icahn School of Medicine at Mount Sinai, New York City, New York, USA; 12 Department of Pathology and Laboratory Medicine, University of Calgary, Calgary, AB Canada; 13 Stefanie Spielman Comprehensive Breast Cancer, The Ohio State University, Columbus, OH USA; 14 Division of Medical Oncology, Comprehensive Cancer Center, The Ohio State University, Columbus, OH USA; 15 Ontario Institute for Cancer Research, Toronto, ON Canada; 16 Edinburgh Cancer Research Centre, Edinburgh, United Kingdom; 17 Department of Laboratory Medicine and Pathobiology, University of Toronto, Toronto, ON Canada; 18 School of Medicine & Dentistry, Kwame Nkrumah University of Science and Technology, Kumasi, Ghana; 19 Department of Child Health, Kwame Nkrumah University of Science and Technology, Kumasi, Ghana; 20 Department of Pathology and Cell Biology, Columbia University Medical Center, New York, New York, USA; 21 Department of Genetics and Genomic Sciences, Icahn School of Medicine at Mount Sinai, New York City, New York, USA; 22 Department of Dermatology, Icahn School of Medicine at Mount Sinai, New York City, New York, USA; 23 Cancer Research UK Cambridge Centre, Cambridge, UK; 24 Lady Davis Institute and Segal Cancer Centre, Jewish General Hospital, Montréal, QC Canada; 25 Program in Cancer Genetics, Departments of Oncology and Human Genetics, McGill University, Montréal, QC Canada; 26 School of Public Health, Loma Linda University, Loma Linda, CA USA; 27 Peace and Love Hospital, Kumasi, Ghana; 28 Kumasi Center for Collaborative Research in Tropical Medicine (KCCR), Kumasi, Ghana; 29 CRUK Edinburgh Centre, University of Edinburgh, Edinburgh, UK; 30 Ghana Statistical Service, Accra, Ghana; 31 Komfo Anoyke Teaching Hospital, Kumasi, Ghana; 32 New York-Presbyterian/Weill Cornell Medical Center, New York, NY USA; 33 National Cancer Institute, Bethesda, MD USA; 34 Westat, Inc, Rockville, MD USA

**Keywords:** Cancer, Breast cancer, Diagnostic markers, Cancer genomics, Molecular medicine

## Abstract

Circulating tumor DNA (ctDNA) sequencing studies could provide novel insights into the molecular pathology of cancer in sub-Saharan Africa. In 15 patient plasma samples collected at the time of diagnosis as part of the Ghana Breast Health Study and unselected for tumor grade and subtype, ctDNA was detected in a majority of patients based on whole- genome sequencing at high (30×) and low (0.1×) depths. Breast cancer driver copy number alterations were observed in the majority of patients.

## Introduction

There is a need to increase diversity in genomic research, particularly in African populations, where more aggressive early-onset breast cancers are diagnosed, and non-invasive methods could provide novel insights for cancer prevention and treatment^1^. This would have both regional and global impacts. Breast cancer incidence is rising in Africa and fast becoming the most common cancer on the continent with a high mortality rate^2^. Technological advances in genomic and bioinformatic analysis of blood-based biomarkers^3,4^ offer opportunities for translational molecular oncology studies in Africa that could inform clinical-decision-making, including precision medicine treatments and new paradigms for prevention, surveillance, diagnosis, and clinical management of cancer.

Much of the literature has focused on ctDNA as an early detection/diagnosis and prognostic tool in high-income countries where it is not yet known whether ctDNA is a more sensitive marker than mammography for early-stage cancer detection^5^. It is also not known whether it can reduce mortality from breast cancer in populations with mammography screening programs. So far, the use of ctDNA as a means for discovery of cancer driver genes has been limited to advanced metastatic tumors where there is also access to solid biopsies and the concentration of ctDNA is above the limit of detection of current sequencing technologies^3^.

We were keen to test if cell-free DNA (cfDNA) sequencing technology could be used to investigate the molecular pathology of cancer in understudied populations such as in Ghana. While it is well established that there is international variation in incidence and mortality rates of breast cancer, the distribution of molecular genetic markers has been challenging to capture due to biases in the collection and processing of tumor tissues^6^. Understanding the distribution of molecular subtypes of breast cancer can inform primary and secondary prevention efforts given that different risk factor associations can help to clarify the natural history of cancer in understudied populations. Here, we wanted to determine if we could capture the molecular genetic landscape of tumors through blood collection, given that public health, primary, and secondary prevention programs are shaped by differences in tumor biology and that cancer etiology is poorly understood in Africa and other low and middle income countries (LMIC).

In addition to understanding molecular pathology, ctDNA could also be a means to improve earlier diagnosis. Analysis of survival across five sub-Saharan countries support that reducing delays to diagnosis and increasing access to available treatments could significantly improve survival outcomes^7^. We recognize that delays to diagnosis is a multifaceted challenge that requires improved education and outreach programs, access to preventive services, and treatment for cancer. We reason that improved points of care for molecular diagnostics such as cfDNA/ctDNA studies might all assist in diagnosing patients quicker and providing them with the appropriate treatments that may be more effective to improve survival.

Whole-genome sequencing of cell-free DNA (WGS-cfDNA) can be used to identify mutations from tumor cells that have shed their DNA into the bloodstream in the form of ctDNA^4,8,9^. Mutations in ctDNA have been studied mostly in populations of European ancestry to investigate utility for diagnosis, screening, early detection, tumor classification, and monitoring responsiveness to treatments^3,4^. With increased investments in emerging technologies and bioinformatics, similar molecular oncology and precision medicine studies might also be possible in Africa^10–12^. To establish a proof-of-concept, we used samples from Ghana, where organized mammography screening programs are lacking and late presentation is common, with the majority (62%) of breast cancer patients are diagnosed with tumors >5 cm in size^13^. We have previously shown that ~64% of incident breast cancers in Ghana were below age 55 years and triple negative breast cancers (TNBCs) were more frequent than in predominantly European populations^1^, where data show mammography screening is less effective^14^.

Knowledge about the genomic landscape of tumors among women in Africa is limited; therefore, we tested whether ctDNA is readily detectable in Ghanaian breast cancer patients. We hypothesized that WGS-cfDNA in African women with breast cancer could reveal somatic alterations^15^ of potential clinical relevance and provide new genomic insights.

## Results

### Circulating tumor DNA fractions of Ghanaian women at the time of breast cancer diagnosis

**Fig. 1:**
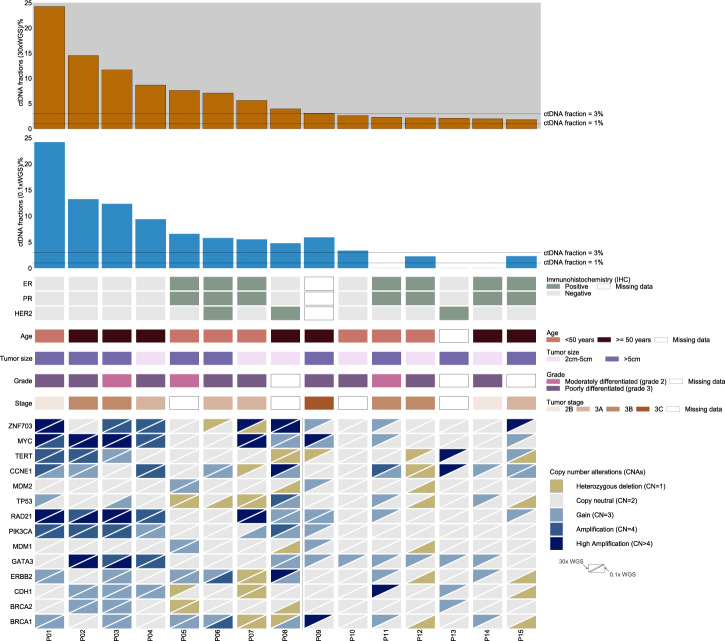
Co-mutation plot showing association between clinical information and genomic characteristics. Patients are represented by the columns ordered by decreasing ctDNA fraction. The top two rows show bar plots of tumor fractions estimated with ichorCNA from cfDNA sequenced with 30× WGS-cfDNA (gold bars) and 0.1× WGS-cfDNA (blue bars) (*n* = 15 patients). The horizontal line across the bar plot shows the limit of detection of ichorCNA for 0.1× (ctDNA fraction = 3%) and a threshold for 30x (ctDNA fraction = 1%) for the detection of ctDNA. Immunohistochemical stains for ER, PR and HER2+, age, and tumor size classification are presented. Copy number gain and loss of selected driver genes in breast cancers are shown in the bottom panel.

The 30× WGS-cfDNA analysis indicated that all 15 breast cancer patients had at least 1% of ctDNA fraction out of total cfDNA (median [IQR] 3.96% [2.22–8.13%]). A comparison of the estimated ctDNA fraction from the 30× cfDNA-WGS with the 0.1× WGS-cfDNA showed high concordance between estimated ctDNA fractions (Pearson *r* = 0.99, *p* = 5.4e−09; Supplementary Fig. 1). Using 0.1× WGS-cfDNA, a minimum of 1% ctDNA fraction was detected in 12 (80%) (95% CI 52%–96%, Fig. 1). Comparisons between ctDNA fractions and clinico-pathological characteristics were made (Supplemental Fig. 2). Due to the small sample size, we were limited in power to detect statistically significant differences. Interestingly, TNBC patients had higher levels of ctDNA compared to non-TNBC patients (*p* = 0.04; two-sided Wilcoxon rank sum test). The four patients with the highest ctDNA fraction were TNBC patients (out of total 5) which is consistent with other populations^16^. Other studies have reported that TNBCs show more aggressive features^17^ including being diagnosed at earlier ages, higher grade, stage, and larger tumor size compared to luminal types^18^. However, we observed that the stage of the non-TNBC patients was similar to that of TNBC patients, and the patient with the highest ctDNA fraction was a stage 2B TNBC patient.

### Copy number profiles of Ghanaian women at the time of breast cancer diagnosis

**Fig. 2:**
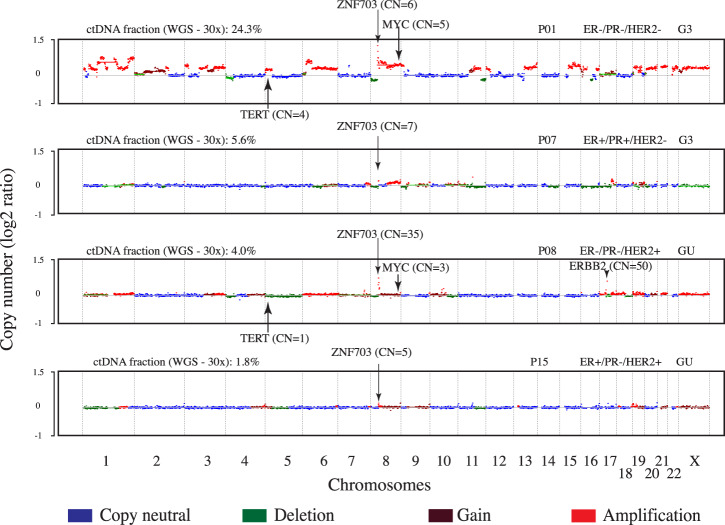
Genome-wide copy number profiles of 4 patients from cfDNA sequenced at 30× WGS. The *x*-axis represents chromosomes (chr1-22:X) and *y*-axis shows log2 copy number. Blue represents copy neutral, i.e., chromosomal loci with standard 2 copies (such as chr3, chr4, and chr5 in P07). Deletions of genomic loci (i.e., chr4p in P01 and P08) are shown in green. Gains (3–4 copies) and amplifications (>4 copies) of chromosomal loci (such as chr8p12 containing ZNF703 genes in P01, P07, P08, and P15) are shown in brown and red colors, respectively. P08 has amplification of the chr17 locus containing *ERBB2*. Arrows indicate the gene and estimated copy number (CN) of the predicted segment by ichorCNA. GU Grade Unknown, G2 moderately differentiated, G3 poorly differentiated, WGS whole-genome sequencing, CN copy number, ER estrogen receptor, PR progesterone receptor, HER2 human epidermal growth factor receptor 2, ctDNA circulating-tumor DNA.

Copy number profiling showed extensive amplification and deletion of multiple chromosomal regions (Fig. 2 and Supplementary Fig. 3) including those containing oncogenes and tumor suppressor genes associated with breast cancer (Fig. 1)^19^. We observed a high frequency (>50%) of copy number gain at three well-known breast cancer loci that contain potential oncogenes (chr8p11.23 [*ZNF703*] *n* = 8, 53.3%; chr8q24.2 [*MYC*] *n* = 9, 60%; chr19q12 [*CCNE1*] *n* = 9, 60%). In The Cancer Genome Atlas (TCGA), chr8p11.23 was amplified in 12.7% of breast tumors from patients of African Ancestry (AA) compared to 11.2% of European Ancestry^19^. We observed high-level amplification (more than four copies) of chr8p11.23 in four (27%, 95% CI 8%–55%) Ghanaian samples.

Focal amplification of chr8p11.23 has been associated with luminal B tumors^20,21^ and late recurrence^22^ in women of European ancestry. The amplification was associated with worse outcomes for patients compared with those who had no change in copy number even after adjustment of stage, grade, and cancer subtype^23^. In our study, the tumors of patients with high amplification of chr8p11.23 regions were observed across the different IHC-defined molecular subtypes of breast cancer. Our data support *ZNF703* as a possible driver gene, which has been suggested to be relevant in the etiology of breast cancer through regulation of both mammary basal and luminal progenitors^21^. Furthermore, *ZNF703* amplification leads to increase in *ZNF703* expression levels, which has been associated with decrease in overall survival for ER positive and luminal B patients^24^. The amplification of another potential driver in chr8p11.23 includes *FGFR1*, which has been associated with worse outcomes in luminal A breast cancers^20^ as well as resistance to endocrine therapy^25^. A recent publication suggests that histone H3 lysine 36 (H3K36) methyltransferase *NSD3*, may be the target of chr8p11.23 amplification and may suggest potential new therapies^26^.

Another recurrent high-level amplification event in our Ghana patient samples was at chr8q24, a region that contains *MYC*. Nine patients (60%) had at least a copy gain (CN ≥ 3) of the chromosomal locus chr8q24. Chr8q24 amplification has been linked to poor prognosis^27^. An increase in copy number was reported in 70–80% of TNBC patients^28^, which was consistent with our data where we observed three (60%) of five TNBC patients harboring high-level amplification of this region. Interestingly, TNBC patients in our sample showed similar genome-wide copy number patterns compared to TNBC patients of European ancestry^29^. These included loci harboring the two telomerase genes *TERC* and *TERT*, implicating upregulation of telomerase activity in breast oncogenesis^30^.

Furthermore, of the two patients classified as HER2+ based on IHC, only one had increased copy number of the region that included *ERBB2* (50 and 2 copies for patients P08 and P13 respectively), consistent with data showing that classification of HER2 status was variable for different quantitative assays^31^.

We compared the ability to predict copy number status for 15 selected breast cancer genes of interest from 0.1× and 30× WGS data (Supplementary Fig. 4). When we applied a cut-off of 3% ctDNA fraction (previously described as the limit of detection for 0.1× WGS of metastatic breast^3^), we observed >46.7% concordance between gene copies from 0.1× WGS and 30× WGS (Supplementary Fig. 4A). Interestingly, patients with >4% ctDNA fraction, with the exception of patient P09, had at least 80% concordance (Supplementary Fig. 4B) between 0.1× WGS and 30× WGS copy number profile for these selected breast cancer genes. Copy number concordance for each gene per patient pair (0.1× and 30× cfDNA-WGS) with >3% ctDNA was at least 70% (Supplementary Fig. 4C). These results suggest that prediction of copy number status for many selected breast cancer genes is potentially feasible from 0.1× WGS of cfDNA in Ghanaian clinics. Furthermore, these results reiterate the current limitations of ctDNA detection in samples with lower tumor fractions^3^.

## Discussion

Our study shows the depth of genomic information that can be obtained by interrogating WGS-cfDNA from plasma samples of breast cancer patients in Ghana. This can reveal copy number alterations and provide more detailed genomic insights into breast cancer in this and other populations with limited resources. Our observation of key alterations having similar frequencies in Ghanaian and AA breast cancer patients suggests that ctDNA may be a useful method for comparative genomic studies of breast cancer as well as for other cancers. In particular, targeted assays for the detection of high copy numbers of *ZNF703*/*FGFR1*/*NSD3* and *MYC* would increase the sensitivity of their detection in the low fractions of ctDNA circulating in the blood. As this amplification is established to be associated with late-stage tumors^23^, further research is needed to determine factors associated with its higher frequency, and in particular, whether it is due to presentation at a late cancer stage or due to other genetic, environmental or lifestyle associated factors.

This study has some limitations. The unavailability of outcomes data did not allow us to compare ctDNA fractions across different outcomes of breast cancer; therefore, further studies are required to explore the clinical utility of ctDNA in Ghanaian populations. Our study could have benefited from somatic variant detection such as insertions (indels) and single nucleotide variants (SNVs). However, from our previous study, we noted that for sufficient statistical power, more than 150x target sequencing coverage is needed to discover these somatic mutations at ctDNA fractions higher than 10%, and higher coverage is needed in samples with lower ctDNA^3^. Therefore, the current depths of sequencing (30× and 0.1× WGS) is insufficient to reliably identify such somatic variants. Hence, future studies could incorporate whole exome sequencing (WES) or high-coverage gene panel sequencing to determine such somatic mutations of clinical relevance. The lack of controls and small sample size did not allow us to determine the specificity and sensitivity of cfDNA-WGS in detecting cancer. Larger datasets and sequencing results from other populations with outcome data will enable determination of whether chromosomal amplifications are enriched in West-African ancestry or a manifestation of adverse effect of cancer, which would be a key question to address in promoting precision medicine in different populations. Future studies to include cancer-free controls will enable us to evaluate the specificity of cfDNA to detect cancer in African-clinical settings.

On the African continent, cancer incidence is rising, mortality rates are high and access to molecular pathology is limited. Non-invasive methods for molecular tumor subtyping could be transformative. The rapid advancement of sequencing technologies and the estimated ctDNA fraction levels (>1%) in this sample of Ghanaian patients in this study indicate that the exploration of alternative methods such as methylation-based cfMedip-seq and panel testing via cfDNA are warranted. These could have high sensitivity (for at least 0.1% ctDNA fraction) and be suitable for patients at the time of diagnosis^3,4,8^. Moreover, the ability to detect tumors with WGS-cfDNA may open up opportunities to test additional advances for sample collection methods (such as a finger-prick blood test^32^). Since all patients had ctDNA fractions higher than 1%, targeted gene panels of high depth (>1000×) will be able to detect nearly all clonal somatic short insertion deletions mutations in the Ghanaian samples^3^. For discovery of new driver genes or mutational signatures detection, 150× WES can be used in cfDNA samples with more than 8% ctDNA fraction. Also, including outcome data will enable the study of cfDNA fraction and copy number as predictive and prognostic biomarker of breast cancer and response to current therapies in Ghana. New and affordable sequencing technologies would allow more facile translation of cancer genomic data to be used at the point of care. Hence, our study shows that an analysis of ctDNA could be a tool for future molecular oncology studies in Africa that merits further investment for cancer etiology, surveillance, molecular oncology and clinical trial studies that are urgently needed to improve cancer survival in Africa.

## Methods

### Patient selection

This project is based on a case-control study of breast cancer conducted in collaboration with three hospitals in Ghana responsible for treating most breast cancers in the country which has been previously described^1,13^; Korle Bu Teaching Hospital (KBTH) in Accra and Komfo Anokye Teaching Hospital (KATH) and Peace and Love Hospital (PLH) in Kumasi. Eligible cases in this study were women 18–74 years of age who were residents of defined catchment areas surrounding Kumasi and Accra for at least one year and who in the preceding year had a lump suspected to be cancer that resulted in either referral for a biopsy at the study hospitals (KBTH, KATH, and PLH) or for clinical care. Within the GBHS study we had identified 15 patients that had duplicate samples which were identified through quality control analysis to identify genetic duplicates for ongoing efforts to identify genetic susceptibility variants. As these patients had multiple plasma samples, and we were not sure if we would be able to detect cfDNA, these samples represented an excellent opportunity to test cfDNA and ctDNA protocols that if successful could be used in the wider collection of over 4000 subjects. These subjects were not selected for any other characteristics and are a random selected set that shows similar demographics and tumor characteristics to the rest of the invasive cases in the study. Demographics and tumor characteristics of the 15 participants selected for the cfDNA study were similar to the overall patient population, with a median age at diagnosis of 49.5 (IQR 44.3–57.8) years.

The study was approved by the Special Studies Institutional Review Board of the National Cancer Institute (Rockville, MD), the Ghana Health Service Ethical Review Committee and institutional review boards at the Noguchi Memorial Institute for Medical Research (Accra, Ghana), the Kwame Nkrumah University of Science and Technology (Kumasi, Ghana), the School of Medical Sciences at Komfo Anokye Teaching Hospital (Kumasi, Ghana) and Westat (Rockville, MD). All participants provided written informed consent.

### Clinical specimens–tumor biopsy

In addition to extracting information from medical records into standardized case abstract forms, study pathologists (Dr. Duggan) performed a centralized histopathology review of H&E sections from biopsy formalin-fixed paraffin-embedded (FFPE) blocks sent to the US (~75% of malignant cases).

### Clinical specimens–blood samples

Venous blood samples were collected using a 10 ml purple top EDTA tube at the time of recruitment, which was usually the time of suspected breast cancer. Purple top tubes were centrifuged and processed within two hours of collection to avoid red blood cell (RBC) contamination of the separated plasma layer. Each collected purple-top tube was kept at room temperature (18–25 °C or 64–77 °F) and processed for a minimum of 15 min at 1500 × *g*/relative centrifugal force (RCF). The top plasma layer was separated from buffy coat, and red blood cells. Three 1.8 ml plasma aliquots were stored using cryovials. Processed plasma specimens were stored at −80 °C freezers. cfDNA was extracted from 2 aliquots of 1.8 ml plasma samples and sequenced at low depth (0.1x ULPS) and at higher depth (30× WGS).

### Tumor characterization (tumor grade, size, stage, and immunohistochemistry)

FFPE blocks received from Ghana were re-embedded prior to obtaining 5 µm H&E sections for pathology review by study pathologists using a standardized form to capture the quality of the specimens and histopathology diagnosis and grade of tumors (See Appendix A, Supplementary Material). IHC data were based on assessment in Ghana as recorded on case abstract forms. A concordance study comparing IHC staining results by Ghanaian pathologists from diagnostic blocks in Ghana and by an NCI pathologist using the study blocks at NCI (i.e., different FFPE blocks from the same patient) showed good agreement for ER (79% agreement based on 87 cases) and HER2 (78% based on 76 cases); however, the agreement for PR was lower (65% based on 86 cases) as previously described^1^.

Tumor size was based on the clinical assessment as previously described^13^. At the time of biopsy, data were recorded by nurses and physicians on the presenting symptoms, number of lumps/masses present, and the approximate size of the lumps/masses from which biopsies were obtained. For the 15 patients included in the cfDNA study, nine patients (60%) presented with tumors larger than 5 cm, and the remaining presented with tumors 2–5 cm^1^. Nine (75%) of 12 patients with pathologic grade data were poorly differentiated. For three (20%) patients, grade information was not available.

Tumor subtypes by immunohistochemistry were defined as previously described^1^. Immunohistochemical (IHC) staining was available for the majority of patients selected for the cfDNA study and was used to classify proxies of molecular subtypes (Fig. 1): Luminal A (ER+ or PR+ and HER2−, *n* = 6, 40%), Luminal B (ER+ or PR+ and HER2+, *n* = 1, 6.7%), HER2-enriched (ER−, PR− and HER2+, *n* = 2, 13%) and triple negative breast cancer (TNBC)/basal (ER−/PR−/HER2−, *n* = 5, 33.3%)^33^ and indeterminate (missing ER, PR, and HER2 IHCs, *n* = 1, 7%).

Of the 15 participants selected, clinical stage was available for 73.3% (*n* = 11) of patients, 13.3% (*n* = 2) were stage 2B, 60% (*n* = 9) were stage 3 (of which 44.4% were 3A, 44.4% were 3B and 11.1% was 3C) and 26.7% (*n* = 4) had no stage information available.

### cfDNA extraction from whole blood

cfDNA extraction, library preparation, and sequencing were conducted at the Broad Institute’s Genomics Services as fee-for-service according to their protocols^3^. Whole blood was collected in EDTA tubes and processed for plasma fractionation within 3 h of blood draw. Blood tubes were centrifuged at 1900 × *g* for 10 min and plasma was transferred to second tube before further centrifugation at 15,000 × *g* for 10 min. Supernatant plasma was stored at −80C until cfDNA extraction. cfDNA was extracted using the QIAsymphony DSP Circulating DNA Kit according to the manufacturer’s instructions, with 6.3 mL of plasma as input and with a 60 μL DNA elution (Qiagen, 2017). Whole blood was collected in EDTA tubes and processed for plasma extraction utilizing two spins. Blood tubes were centrifuged at 1900 × *g* for 10 min and plasma was transferred to a second tube before further centrifugation at 15,000 × *g* for 10 min. Supernatant plasma was stored at −80C until cfDNA extraction. The preferred starting input volume is 6.3 mL plasma, if a sample did not meet this input phosphate-buffered saline (PBS) was added. cfDNA was extracted using the QIAsymphony DSP Circulating DNA Kit according to the manufacturer’s instructions. This was a magnetic-particle technology-based chemistry used in conjunction with the QIAsymphony SP instrument manufactured by Qiagen. The cfDNA was bound to magnetic particles. The particle-bound cfDNA was separated from the solution using a covered magnetic rod head. Several wash steps followed to eliminate debris and protein residues from the sample. The machine finished with a 60 μL cfDNA elution.

### Library construction

Initial DNA input was normalized to be within the range of 25–52.5 ng in 50 μL of TE buffer (10 mM Tris HCl 1 mM EDTA, pH 8.0) according to picoGreen quantification. Library preparation was performed using a commercially available kit provided by KAPA Biosystems (KAPA HyperPrep Kit with Library Amplification product KK8504) and IDT’s duplex UMI adapters. Unique 8-base dual index sequences embedded within the p5 and p7 primers (purchased from IDT) were added during PCR. Enzymatic clean-ups were performed using Beckman Coultier AMPure XP beads with elution volumes reduced to 30 µL to maximize library concentration.

### Post library construction quantification and normalization

Library quantification was performed using the Invitrogen Quant-It broad range dsDNA quantification assay kit (Thermo Scientific Catalog: Q33130) with a 1:200 PicoGreen dilution. Following quantification, each library was normalized to a concentration of 35 ng/µL, using Tris-HCl, 10 mM, pH 8.0.

### Library pool creation for ultra-low pass sequencing

In preparation for the sequencing of the ultra-low pass libraries (ULP), approximately, 4 µL of the normalized library was transferred into a new receptacle and further normalized to a concentration of 2 ng/µL using Tris-HCl, 10 mM, pH 8.0. Following normalization, up to 95 ultra-low pass WGS samples were pooled together using equivolume pooling. The pool was quantified via qPCR and normalized to the appropriate concentration to proceed to sequencing.

### Cluster amplification and sequencing

Cluster amplification of library pools was performed according to the manufacturer’s protocol (Illumina) using Exclusion Amplification cluster chemistry and HiSeq X flowcells. Flowcells were sequenced on v2 Sequencing-by-Synthesis chemistry for HiSeq X flowcells. The flowcells were then analyzed using RTA v.2.7.3 or later. Each pool of ultra-low pass whole-genome libraries was run on one lane using paired 151 bp runs. For 30× WGS, the same approach used for ULP Unique Molecular Identifiers (UMI) were adopted. We added extra lanes of sequencing to the ULP library to achieve 30x.

### Next-generation sequencing (NGS) data preprocessing

Raw reads in the form of.fastq files were preprocessed using the GATK best practices for NGS data (Fig. 1). This involves mapping of raw reads to human reference genome (Hg19/GRCh37) using Burrows-Wheeler Aligner (BWA-MEM) algorithms^34^ to generate a Sequence Alignment/Map (SAM) file of mapped reads. Sam files were converted to a binary alignment format (BAM) file of aligned reads^35^.

Duplicate reads from polymerase chain reaction (PCR) during the library preparation step of sequencing were removed with “gatk markduplicates” software to allow us to confidently detect which DNA segments are amplified or delete due to carcinogenesis^36^. The base quality score recalibration (BQSR) step which employs machine learning algorithms (GATK BaseRecalibrator tool) to model the systematic errors from the sequencing machine and subsequent correction with gatk ApplyBQSR tool were done to produce an analysis-ready-bam file for tumor fraction estimation, ploidy, and copy number aberrations using ichorCNA software. Quality control (QC) including fragment size distribution was evaluated (Supplementary Fig. 5).

All the data preprocessing steps were carried out in Terra (https://app.terra.bio/), a cloud-based analysis platform with publicly available NGS workflows developed by the Broad Institute in collaboration with Google Cloud.

### Tumor fraction detection and copy number profiles prediction

To estimate the tumor fraction (cell-free DNA originating from neoplastic cells) and copy number alterations, the ichorCNA software was used^3^. The software accepts an analysis-ready-bam file as input and simultaneously estimates tumor fractions and copy number profiles through a three-step process (1) dividing the genome into non-overlapping windows of specific length and computing read coverage within each bin, (2) read normalization; correcting for Guanine-Cytosine content, replication timing, mappability of reads, (3) tumor fraction estimation and CNA prediction; implemented using Hidden Markov Models (HMM) segmentation algorithm to find neighboring bins as genomic regions that are amplified or deleted together and the expectation-maximization algorithms to estimate the parameters of the model.

For 0.1x ULPS analysis, ichorCNA (v0.2.0) release version (https://github.com/broadinstitute/ichorCNA) was used with default settings (https://github.com/broadinstitute/ichorCNA/blob/master/scripts/snakemake/config/config.yaml).

Briefly, the configuration settings are the following:

A 1MB bin size was used to compute coverage. The following resources, Guanine + Cytosine (GC) score, and mappability all in 1MB.wig file format for hg19 were used to normalize reads when running ichorCNA. Centromere resource files based on UCSC (GRCh37.p13) were downloaded from UCSC and used to mask repetitive sequences in the human genome that the aligner may have erroneously mapped to. A panel of normals (PoN) from 27 healthy donors provided in the ichorCNA package as a resource was used. The threshold applied for the minimum mapping score was 0.75. To control segmentation, which will ultimately affect sensitivity, the transition probabilities were set as ichorCNA_txnE = 0.99 and ichorCNA_txnStrength=100. The non-tumor fraction parameter restart values of 0.5, 0.6, 0.7, 0.8, 0.9, 0.95 were used. Tumor ploidy restart values of 2 and 3 were set as parameters. Subclonal copy number (1,3) was set as part of the parameters. The maximum copy number value of 5 and Student’s-*t* likelihood model were set as parameters. Solutions for each parameter restart were ranked by the log-likelihood value, and only solutions were included if the following criteria were met: Maximum fraction of genome accounted as subclonal was <0.5, the maximum fraction of genome in subclone was <0.7. Optimal solutions were selected based on the log-likelihoods. Manual inspection of these solutions were performed to confirm the results.

In the 30× WGS analysis, an updated version of ichorCNA (https://github.com/GavinHaLab/ichorCNA) was used with custom configurations to analyze deeper WGS data and to increase the sensitivity needed to be able to detect low tumor fractions. Parameters used for analysis are stored as config files and are publicly available on GitHub (https://github.com/sahuno/cfDNA_Ghana_pilot_GBHS/tree/master/scripts/ichorCNA_configs/WGS_30x_GBHS_ichorCNA_config).

The key distinguishing steps here are that;

1. mappability was accounted for
2. ichorCNA_txnStrength was decreased from default value of 10,000 to 100 to employ more segments leading to higher sensitivity.
3. The ichorCNA_txnE used was 0.99 to increase sensitivity.
4. The –minSegmentBins was reduced from 50 to 20
5. The –altFracThreshold was reduced 0.05 to 0.01

### Annotation of somatic copy number variants

To annotate copy number alterations, Ensembl genes^37^ (human reference genome GRCh37.p13) with coordinates (Chromosomes 1:22, X, Y, without patches) were retrieved from Ensembl legacy website http://grch37.ensembl.org/biomart/martview/2c80f6edb70f976fa9dbcdc5127d8cf0 (accessed date; Friday, February 14, 2020) programmatically with R package Biomart^37^.

The following attributes were used to query reference genes from Ensembl, Gene stable ID, Chromosome/scaffold name, Gene start (bp), Strand (+/−), Gene end (bp), Gene name, Human Genome Organization (HUGO) Gene Nomenclature Committee (HGNC) symbol, Gene type. The queried Ensembl gene list with coordinates and metadata and copy number segments were converted to Genomic ranges object using the ‘GenomicFeatures‘ package to allow complete overlap between unique genes and copy number segments. The Genomic ranges object is a type of object in R used to conveniently store gene information (chromosome, start position, end position, strand) and other metadata of the gene (gene name, gene ID, HGNC symbol, etc.). For simplicity, the HGNC symbols (unique and meaningful naming schemes for known human genes) of annotated genes were used throughout the analysis^38^.

The frequency of copy number changes was calculated for a subset of genes known to be recurrently altered in chromosomal segments extracted from TCGA (cbioportal, pan cancer breast study) sorted according to GISTIC scores/q-value. GISTIC (Genomic Identification of Significant Targets in Cancer) is an algorithm for detecting possible cancer driver genes in copy number profiles by evaluating the frequency and amplitude of the events (loss and/or gain of genomic regions)^39^. Hence the associated GISTIC *q*-value and score was the metric used for accepting possible drivers^39^.

To determine the frequency of amplification of known breast cancer genes between African and European ancestry, the TCGA breast cancer (pan-cancer) via cbioportal (https://www.cbioportal.org/) was used.

### Metrics for determining concordance between 0.1× WGS and 30× WGS for copy number detection

To determine whether similar copy number alterations could be detected by sequencing cfDNA at 0.1× WGS and 30× WGS, we developed two metrics to quantitatively test this out. Using a list of 15 known cancer genes (as shown in CoMut plot) we calculated (1) copy number concordance per gene level (Eq. 1) (2) copy number concordance at the cohort level (Eq. 2).

In generating copy number concordance per gene level, for each patient, we assigned the value ‘1‘ for a gene copy number match between 0.1× and its 30× WGS. A mismatch was assigned a “0”. For simplicity, amplifications and high amplifications were recategorized as copy gain and only gain, loss, neutral were used for this exercise. We then calculated the sum of matches and mismatches for each gene across our samples with at least 3% ctDNA fraction and divided by the total number of samples with ≥3% ctDNA fraction and expressed as a percentage. The 3% ctDNA fraction cut-off was used following the observation that copy number events were more similar as ctDNA fraction increased. Similar observations were found in benchmark exercises using ichorCNA among metastatic breast and prostate cancer^3^.

1 $$GeneX = \frac{\begin{array}{l}1(patient\_1\_GeneX\_01xULPS = = patient\_1\_GeneX\_30xWGS) + \\ 0(patient\_2\_GeneX\_01xULPS \ne patient\_2\_GeneX\_30xWGS) + . \ldots \\ 1(patient\_n\_GeneX\_01xULPS = = patient\_n\_GeneX\_30xWGS) + \\ 0(patient\_n\_GeneX\_01xULPS \ne patient\_n\_GeneX\_30xWGS)\end{array}}{{total\,number\,of\,patients\,(cfDNA\,fraction \ge 3\% )in\,cohort}} \ast 100\%$$

To determine concordance at the cohort level, copy number match of each gene from sample sequenced at 0.1× and its pair sequenced at 30× WGS was assigned a ‘1‘ whereas for a mismatch, a value of ‘0‘ was assigned (Eq. 2). Amplifications and high amplifications were recategorized as copy gain, for simplicity, therefore only copy gain, loss, and neutral were used. For each patient we summed up the number matches and mismatches for all genes, divided by the total number of genes (in the CoMut plot) and expressed as percentage. We showed that patients with higher ctDNA fractions had higher percentage of concordance for copy number detection between 0.1× and 30× WGS.

2 $$patient\_1\_01xULPS\& 30xWGS = \frac{\begin{array}{l}1(patient\_1\_GeneA\_01xULPS = = patient\_1\_GeneA\_30xWGS) + \\ 0(patient\_1\_GeneB\_01xULPS \ne patient\_1\_GeneB\_30xWGS) + \ldots .\\ \left. {1(patient\_1\_GeneX\_01xULPS = = patient\_1\_GeneX\_30xWGS)} \right)\end{array}}{{total\,number\,of\,genes\,of\,{\it{interest}}}} \ast 100\%$$

$$patient\_n\_01xULPS\& 30xWGS = \frac{\begin{array}{l}1(patient\_n\_GeneA\_01xULPS = = patient\_n\_GeneA\_30xWGS) + \\ 0(patient\_n\_GeneB\_01xULPS \ne patient\_n\_GeneB\_30xWGS) + \ldots .\\ \left. {1(patient\_n\_GeneX\_01xULPS = = patient\_n\_GeneX\_30xWGS)} \right)\end{array}}{{total\,number\,of\,genes\,of\,{\it{interest}}}} \ast 100\%$$

### Statistical analysis

Median and interquartile (IQR) of ctDNA fractions were calculated and compared with clinico-pathological variables (age, grade, stage, and subtypes. Pearson correlation analysis was used to calculate the Pearson correlation coefficient between ctDNA fractions from cfDNA sequenced at 30× WGS and 0.1× WGS. A two-tailed Mann–Whitney *U* Test was used to calculate the difference in ctDNA fractions for ER+ vs ER−, <50 years vs 50 years and above, grade 2 vs grade 3 tumors and medium vs large-sized tumors. The two-sided Wilcoxon signed-rank test was used to calculate significant differences between ctDNA fractions between TNBCs and Non-TNBC breast cancers. *P* values <0.05 were considered significant.

## Supplementary information

Supplementary materials

Supplementary Table 1

## Data Availability

Genomic sequencing data are available from the dbGaP repository under accession phs002387.v1.p1. These data are available for breast cancer and other adult disease research only.

## Code availability

Code for ichorCNA v0.2.0 used to analyze 0.1× WGS-cfDNA sequence reads can be accessed at https://github.com/broadinstitute/ichorCNA. Analysis of 30× WGS-cfDNA data used an updated version of ichorCNA and can be accessed at https://github.com/GavinHaLab/ichorCNA [commit b2bbce0, June 22, 2020].
