# Supplementary material for: Circulating tumor DNA is readily detectable among Ghanaian breast cancer patients supporting non-invasive cancer genomic studies in Africa
Source: NPJ Precis Oncol. 2021 Sep 17;5:83. doi: 10.1038/s41698-021-00219-7 (PMC8448727; doi:10.1038/s41698-021-00219-7)
Supplement: Supplementary file 1 — Supplementary materials [file 41698_2021_219_MOESM1_ESM.pdf]

Supplementary materials for

Circulating tumor DNA is readily detectable among Ghanaian breast cancer patients supporting non-invasive cancer genomic studies in Africa

Samuel Terkper Ahuno[1,2], Anna-Lisa Doebley[3,4], Thomas U. Ahearn[5], Joel Yarney[6], Nicholas Titiloye[7], Nancy Hamel[8], Ernest Adjei[7], Joe-Nat Clegg-Lamptey[6,9], Lawrence Edusei[6], Baffour Awuah[7], Xiaoyu Song[10,11], Verna Vanderpuye[6], Mustapha Abubakar[5], Maire Duggan[12], Daniel G. Stover[13,14], Kofi Nyarko[9], John M.S. Bartlett[15,16,17], Francis Aitpillah[7,18], Daniel Ansong[19], Kevin L. Gardner[20], Felix Andy Boateng[7], Anne M. Bowcock[2,11,21,22], Carlos Caldas[23], William D. Foulkes[8,24,25], Seth Wiafe[26], Beatrice Wiafe-Addai[27], Montserrat Garcia-Closas[5], Alexander Kwarteng[1,28], Gavin Ha[4*^], Jonine D. Figueroa[5,29*^], Paz Polak[2,11,21*^] , on behalf of the Ghana Breast Health Study Team

*These authors jointly supervised this work
^corresponding authors: gha@fredhutch.org, Jonine.figueroa@ed.ac.uk. paz.polak@mssm.edu

BSI ID: TBR______________________________ REVIEWER:______________________ DATE REVIEWED:_______________________

Technical Preparation	Tissue Adequacy	Tumor Adequacy
1a. Satisfactory .... 1 [Go to 2a]<br>Limited .... 2 [Go to 1b]<br>Unsatisfactory .... 3 [Go to 1b]	2a. Satisfactory .... 1 [Go to 3a]<br>Limited .... 2 [Go to 2b]<br>Unsatisfactory .... 3 [Go to 2b]	3a. Satisfactory .... 1<br>Limited .... 2 [Go to 3b]<br>Unsatisfactory .... 3 [Go to 3b]
1b. Circle all that apply:<br>Missing slide ... 1 Cover slipping issues ... 4<br>Broken slide .... 2 Other .... 5<br>Folds .... 3 Specify ____________	2b. Circle all that apply:<br>Crushed (Partial) .... 1 Fixation issues (Partial) .... 5<br>Crushed (Complete) ... 2 Fixation issues (Complete) .... 6<br>Necrotic (Partial) .... 3 Other .... 7<br>Necrotic (Complete) ... 4 Specify _________________	3b. Circle all that apply:<br>No tumor .... 1 Necrotic (Complete) .... 6<br>Small tumor volume ... 2 Fixation issues (Partial) .... 7<br>Crushed (Partial) .... 3 Fixation issues (Complete) .. 8<br>Crushed (Complete) .. 4 Other .... 9<br>Necrotic (Partial) .... 5 Specify ________________

4a. Is Invasive cancer present: Yes .... 1 [Go to 4b and 4c]
No .... 2 [Go to 5a]

4b. **Invasive Diagnosis** (Circle all that apply)

Type	Classification
Invasive carcinoma of no special type (NST)	8500/3
Pleomorphic carcinoma	8522/3
Carcinoma with osteoclast-like stromal giant cells	8035/3
Carcinoma with choriocarcinomatous features	
Carcinoma with melanotic features	
Invasive lobular carcinoma	8520/3
Classic lobular carcinoma; Pleomorphic lobular carcinoma	
Solid lobular carcinoma; Tubulolobular carcinoma	
Alveolar lobular carcinoma; Mixed lobular carcinoma	
Tubular carcinoma	8211/3
Cribriform carcinoma	8201/3
Mucinous carcinoma	8480/3
Carcinoma with medullary features	
Medullary carcinoma	8510/3
Atypical medullary carcinoma	8513/3
Invasive carcinoma NST with medullary features	8500/3
Carcinoma with apocrine differentiation	
Carcinoma with signet-ring-cell differentiation	
Invasive micropapillary carcinoma	8507/3
Metaplastic carcinoma of no special type	8575/3
Low-grade adenosquamous carcinoma	8570/3
Fibromatosis-like metaplastic carcinoma	8572/3
Squamous cell carcinoma	8070/3
Spindle cell carcinoma	8032/3
Metaplastic carcinoma with mesenchymal differentiation	
Chondroid differentiation	8571/3
Osseous differentiation	8571/3
Other types of mesenchymal differentiation	8575/3
Mixed metaplastic carcinoma	8575/3
Myoepithelial carcinoma	8982/3
Epithelial-myoepithelial tumors	
Adenomyoepithelioma with carcinoma	8983/3
Adenoid cystic carcinoma	8200/3
Rare types	
Carcinoma with neuroendocrine features	
Neuroendocrine tumor, well-differentiated	8246/3
Neuroendocrine carcinoma poorly differentiated (small cell carcinoma)	8041/3
Carcinoma with neuroendocrine differentiation	8574/3
Secretory carcinoma	8502/3
Invasive papillary carcinoma	8503/3
Acinic cell carcinoma	8550/3
Mucoepidermoid carcinoma	8430/3
Polymorphous carcinoma	8525/3
Oncocytic carcinoma	8290/3
Lipid-rich carcinoma	8314/3
Glycogen-rich clear cell carcinoma	8315/3
Sebaceous carcinoma	8410/3

4c. Grade: Grade 1 .... 1 Grade 3 .... 3 Not applicable ... 5
Grade 2 .... 2 Unable to grade ... 4

4d. Number of invasive cancer pieces: __________

4e. Number of tissue pieces present: __________

5a. Are non-invasive precursor lesion(s) present: Yes .... 1 [Go to 5b]
No .... 2 [Go to 6a]

5b. **Non-invasive Precursor Lesions Diagnosis** (Circle all that apply)

Type	Classification
Precursor lesions	
Ductal carcinoma in situ	8500/2
Lobular neoplasia	
Lobular carcinoma in situ	
Classic lobular carcinoma in situ	8520/2
Pleomorphic lobular carcinoma in situ	8519/2*
Atypical lobular hyperplasia	
Intraductal proliferative lesions	
Usual ductal hyperplasia	
Columnar cell lesions including flat epithelial atypia	
Atypical ductal hyperplasia	
Papillary lesions	
Intraductal papilloma	8503/0
Intraductal papilloma with atypical hyperplasia	8503/0
Intraductal papilloma with ductal carcinoma in situ	8503/2*
Intraductal papilloma with lobular carcinoma in situ	8520/2
Intraductal papillary carcinoma	8503/2
Encapsulated papillary carcinoma	8504/2
Encapsulated papillary carcinoma with invasion	8504/3
Solid papillary carcinoma	
In situ	8509/2
Invasive	8509/3

6a. Are benign lesions present: Yes .... 1 [Go to 6b]
No .... 2 [End]

6b. **Benign Lesion Diagnosis** (Circle all that apply)

No pathological abnormality	1
Abscess +/- organization	2
Scar	3
Cyst/Cystically dilated duct +/- apocrine metaplasia	4
Ruptured Cyst/Cystically dilated duct +/- macrophage reaction	5
Fat necrosis	6
Stromal fibrosis	7
Stromal calcification	8
Pseudolactational changes, hyperplasia	9
Fibroadenoma	10
Sclerosing adenosis	11
Radial scar	12
Tubular adenoma	13
Intramammary lymph node	14
Granulomatous inflammation	15
Other ____	16

6c. Diagnosis Not Possible 1

Appendix A: Hematoxylin and eosin (H&E) pathology review form

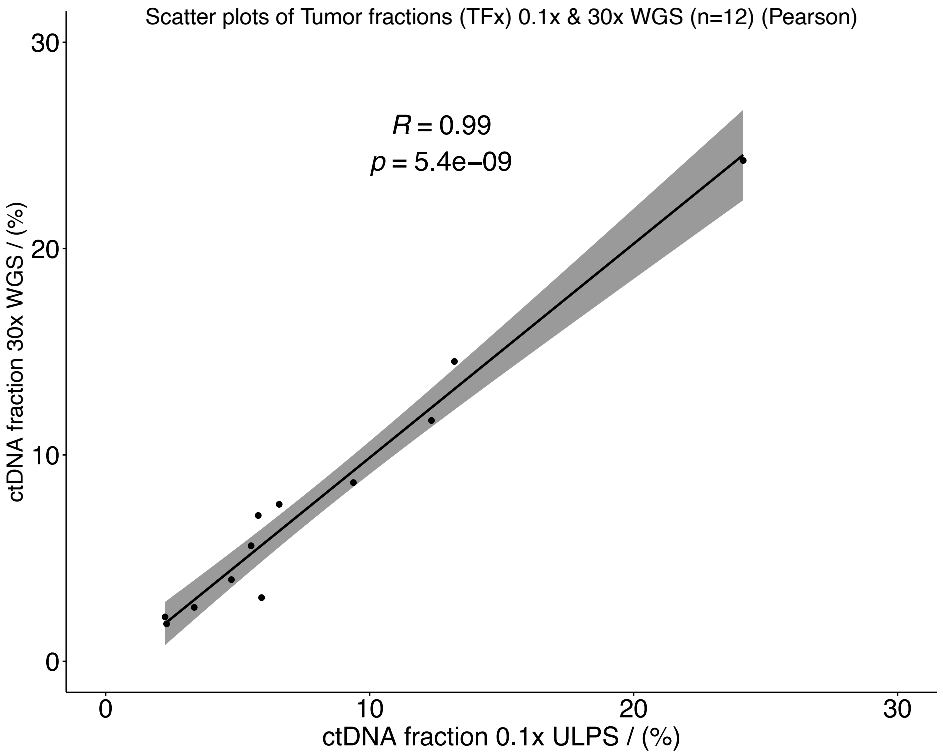

Supplementary Figure 1: Scatter plot of ctDNA fractions (Pearson correlation) The y-axis represents ctDNA fraction from 15 plasma samples sequenced at 30x WGS in percentages. The x-axis repesents ctDNA fractions (in percentages) from the same 15 Ghanaian women at the 0.1x Ultra Low Pass Sequencing (ULPS)

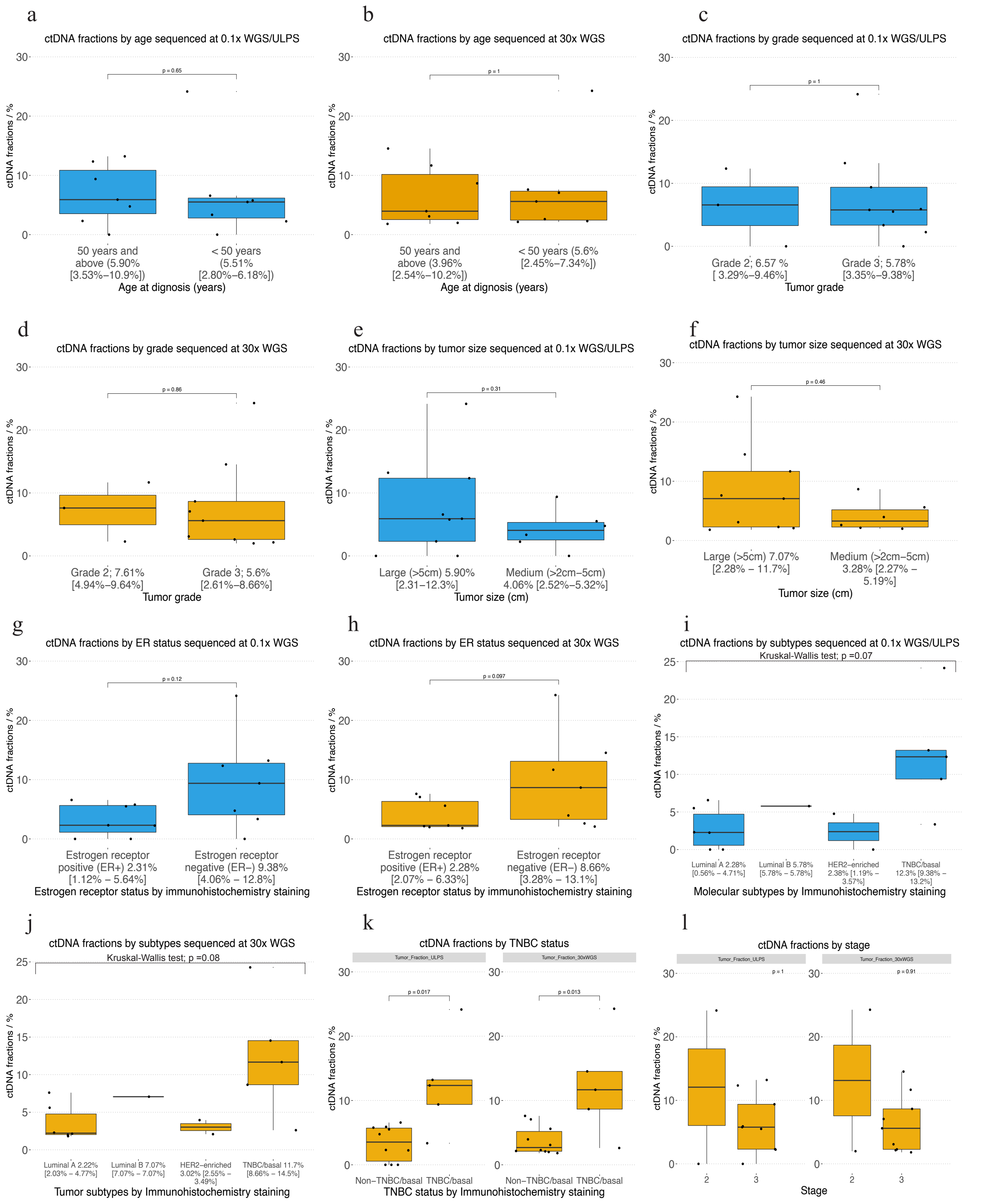

Supplementary Figure 2: ctDNA fractions and clinico-pathological characteristics. a) ctDNA fractions (0.1x WGS by age b) ctDNA fractions (30x WGS) by age c) ctDNA fractions (0.1x WGS) by grade d) ctDNA fractions (30x WGS) by grade e) ctDNA fractions (0.1x WGS) by tumor size f) ctDNA fractions (30x WGS) by tumor size g) ctDNA fractions (0.1x WGS) by ER status h) ctDNA fractions (30x WGS) by ER status i) ctDNA fractions (0.1x WGS) by subtypes j) ctDNA fractions (30x WGS) by subtypes k) ctDNA fractions by TNBC status l) cfDNA fraction by tumor stage. Statistical test in panels a-h, k & l was performed with two-tailed Mann-Whitney U Test. A two-sided Wilcoxon signed-rank test was used for panels i & j.

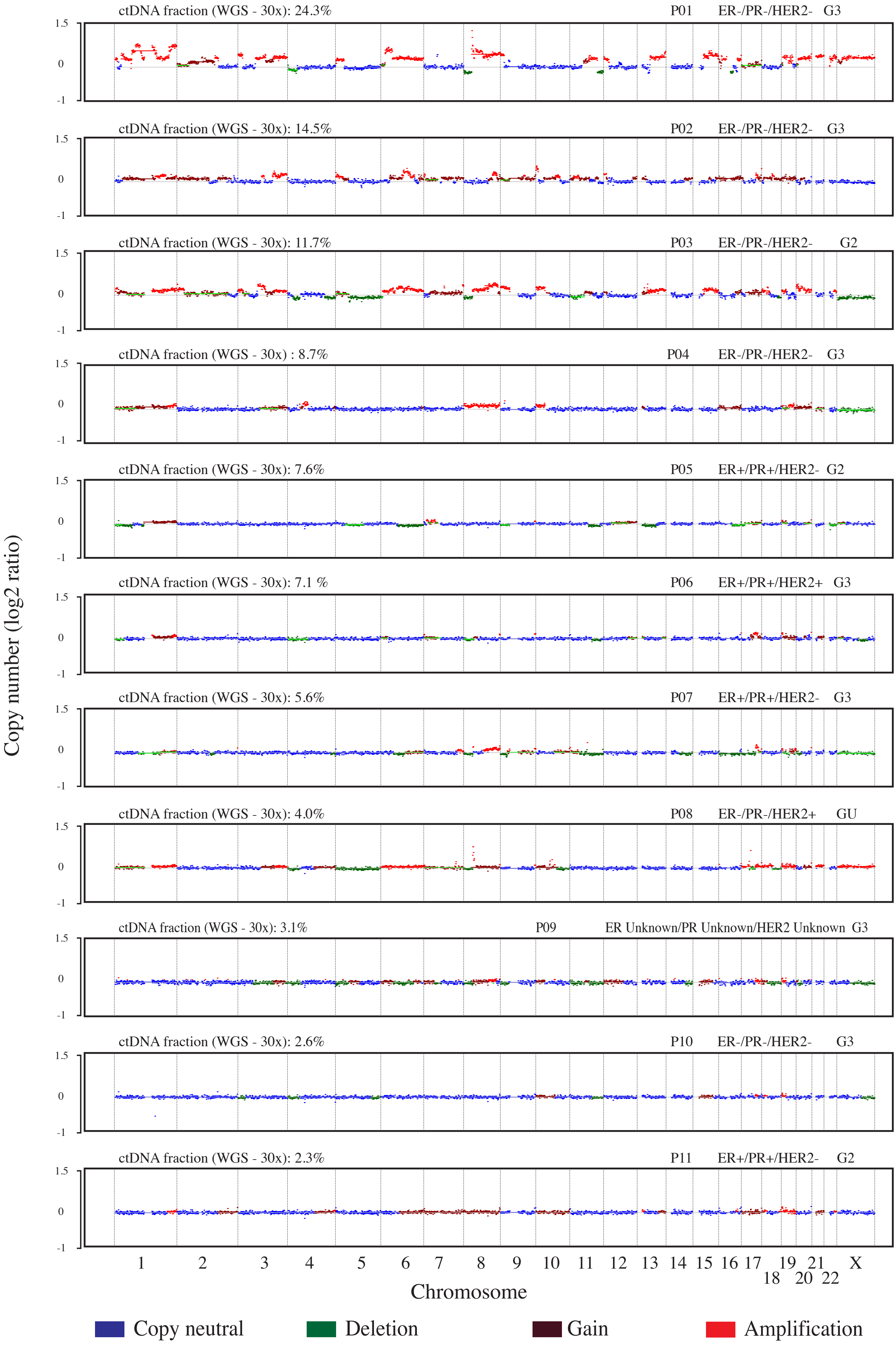
ctDNA fraction (WGS - 30x): 24.3% P01 ER-/PR-/HER2- G3
ctDNA fraction (WGS - 30x): 14.5% P02 ER-/PR-/HER2- G3
ctDNA fraction (WGS - 30x): 11.7% P03 ER-/PR-/HER2- G2
ctDNA fraction (WGS - 30x) : 8.7% P04 ER-/PR-/HER2- G3
ctDNA fraction (WGS - 30x): 7.6% P05 ER+/PR+/HER2- G2
ctDNA fraction (WGS - 30x): 7.1 % P06 ER+/PR+/HER2+ G3
ctDNA fraction (WGS - 30x): 5.6% P07 ER+/PR+/HER2- G3
ctDNA fraction (WGS - 30x): 4.0% P08 ER-/PR-/HER2+ GU
ctDNA fraction (WGS - 30x): 3.1% P09 ER Unknown/PR Unknown/HER2 Unknown G3
ctDNA fraction (WGS - 30x): 2.6% P10 ER-/PR-/HER2- G3
ctDNA fraction (WGS - 30x): 2.3% P11 ER+/PR+/HER2- G2
1.5
0
-1
Copy number (log2 ratio)
1 2 3 4 5 6 7 8 9 10 11 12 13 14 15 16 17 18 19 20 21 22 X
Chromosome
Copy neutral
Deletion
Gain
Amplification

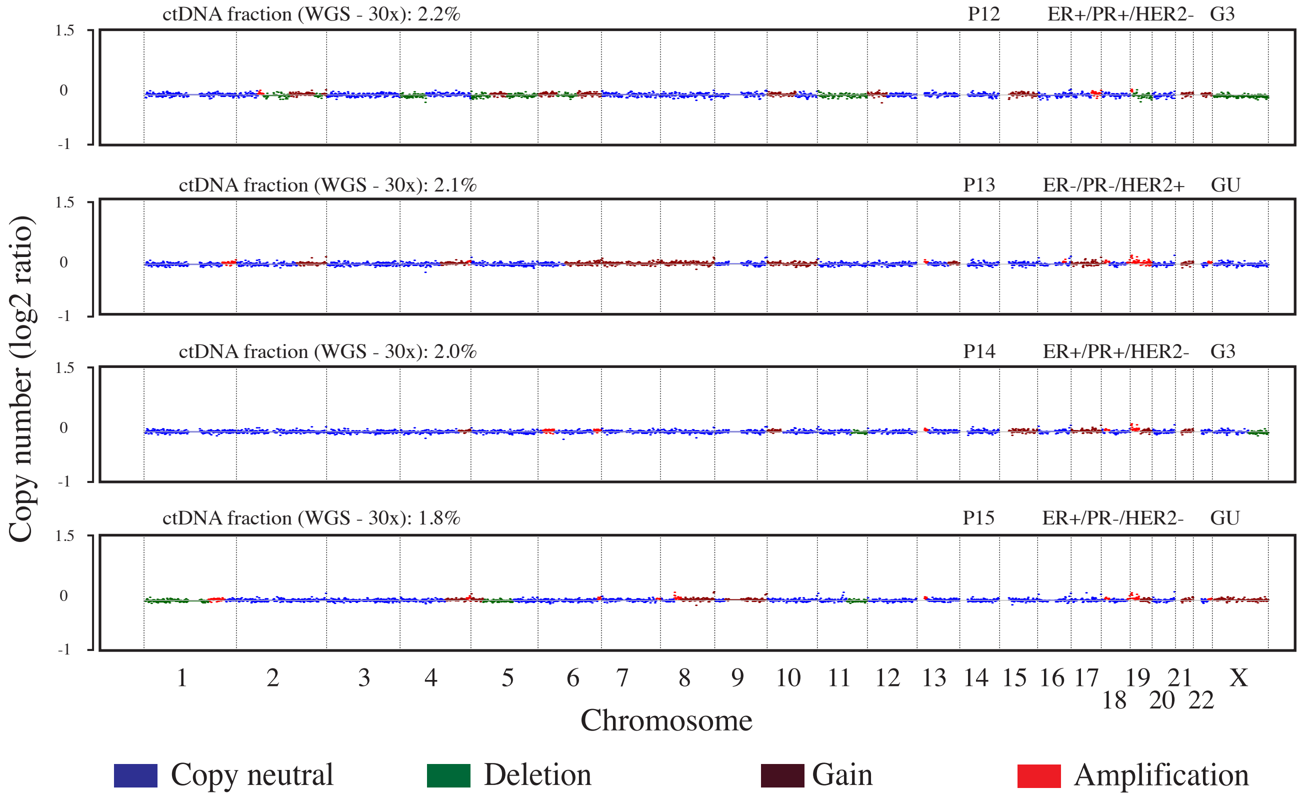

Supplementary Figure 3: Genome-wide copy number profiles of all 15 patients from cfDNA sequenced at 30x Whole Genome Sequencing (WGS). Genome-wide copy number profiles of the fifteen (15) cases arranged in descending order of tumor fractions. The y-axis corresponds to log2 copy ratio estimated by ichorCNA and x-axis are chromosomes (chr1-22 and chrX). Patient IDS, tumor grade, Immunohistochemistry stains, tumor fractions are on top of each plot. GU- Grade Unknown, G2- moderately differentiated, G3-poorly differentiated, WGS- whole genome sequencing, CN-copy number, ER- estrogen receptor, PR-progesterone receptor, HER2 – human epidermal growth factor receptor 2, ctDNA- circulating-tumor DNA

a

b

c

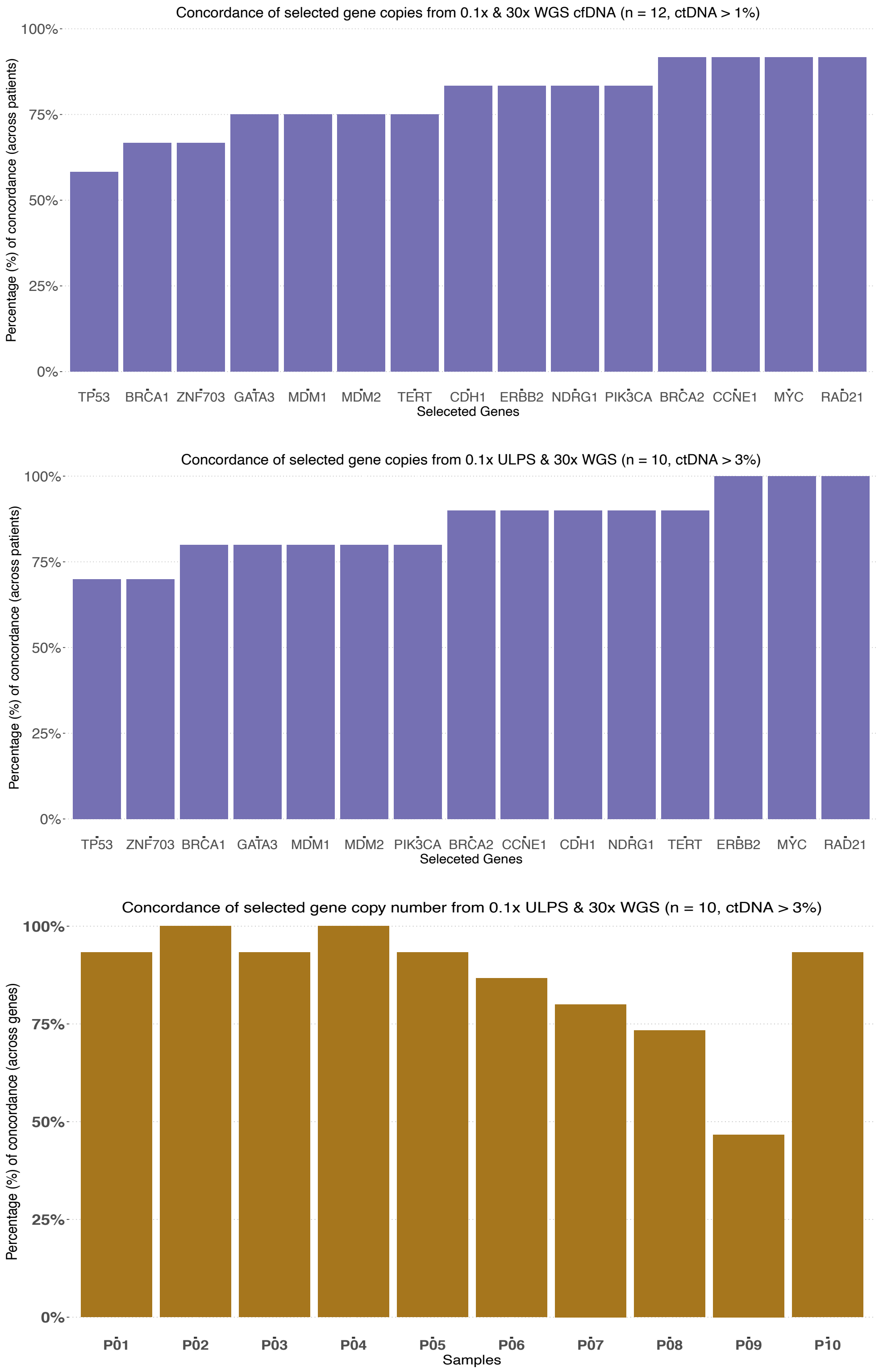

Supplementary Figure 4: Concordance between 0.1x & 30x WGS for copy number detection. a) Concordance of selected gene copies from 0.1x ULPS & 30x WGS (n = 10, ctDNA > 1%) [across all patients] b) Concordance of selected gene copies from 0.1x ULPS & 30x WGS (n = 10, ctDNA > 3%) [across all patients] c) Concordance of selected gene copies from 0.1x ULPS & 30x WGS (n = 10, ctDNA > 3%) [across all genes]

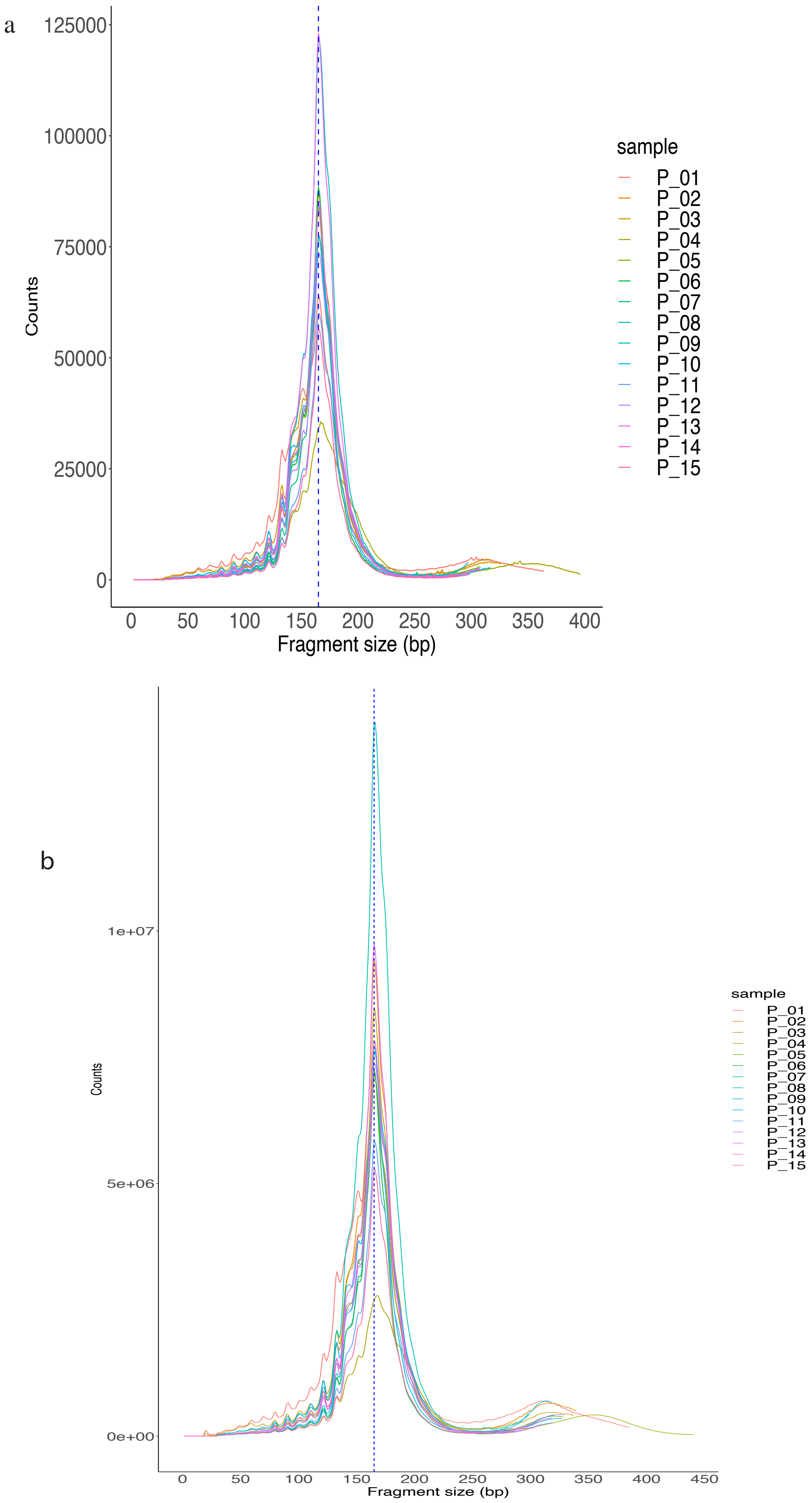

Supplementary Figure 5: Fragment size distribution of cfDNA
A) 0.1x WGS-cfDNA fragment size distribution B) 30x WGS-cfDNAb fragment size distribution
